# Research on hydrogen leakage and diffusion mechanism in hydrogenation station

**DOI:** 10.1038/s41598-023-50917-4

**Published:** 2024-02-09

**Authors:** Zenggang Zhang, Mingheng Shang

**Affiliations:** 1 School of Thermal Engineering, Shandong Jianzhu University, Jinan, 250101 Shandong China; 2 Shandong Provincial Architecture Design & Research Institute CO., Ltd., Jinan, 250101 Shandong China

**Keywords:** Power stations, Fluid dynamics

## Abstract

As a clean, efficient and sustainable energy carrier, hydrogen energy has been accepted as one of the main directions of future energy development. In this paper, a hydrogenation station providing compressed hydrogen outside was adopted as the research object. Based on finite element method and virtual nozzle model, the influence of leakage of main equipment in hydrogenation station on the distribution of combustible hydrogen was investigated, including hydrogen storage tank group, tube trailer, compressor chamber and hydrogenator. The results showed that the shape and volume of the combustible hydrogen cloud generated by the leak were influenced by obstacles, hydrogen storage pressure, and wind velocity. The disturbance of external wind and the decrease in hydrogen storage pressure will have a positive impact on the reduction of leaked volume. The diffusion of combustible hydrogen clouds can be exacerbated by complex structure of obstacles, while partition wall in the adopted hydrogenation station model can limit the combustible hydrogen cloud in the process area. These conclusions can provide guidance and reference for the risk prevention measures of hydrogenation station.

## Introduction

Hydrogen energy, as a clean, efficient, and sustainable energy carrier, has garnered widespread recognition as a pivotal focal point for future energy development^1–3^. Promoting the utilization of hydrogen energy is beneficial for reducing carbon emissions, protecting the environment, which has environmental and social benefits. As the infrastructure to provide hydrogen for hydrogen fuel cell vehicles, the hydrogenation station becomes an indispensable part of hydrogen energy utilization. However, due to the characteristics of hydrogen, such as a low minimum ignition energy (about 0.02 MJ)^4^, a wide range of explosion limit (4%∼75%)^5–7^, the safety of hydrogenation has been always questioned. These factors can easily lead to combustion and explosion accidents involving hydrogen, which made the development of hydrogen energy system restricted^8–10^. Therefore, the analysis and research of the influencing factors related to hydrogen leakage, combustion, and explosions in hydrogenation stations hold significant theoretical and practical importance^11^. This research is crucial for enhancing safety and expediting the healthy development of the hydrogen energy industry.

Relevant scholars have carried out numerous theoretical and experimental researches on the hydrogenation station to reveal the general rules of hydrogen leakage and explosion. De Stefano et al.^12^ studied the impact of leakage location, leakage speed, and obstacles on hydrogen leakage accidents in confined spaces through experimental methods. The results show that the impact of leakage speed on the distribution of hydrogen concentration in confined spaces is greater than the impact of leakage location. The leaked hydrogen encountering obstacles will cause a loss of kinetic energy and increase the hydrogen concentration gradient in the upper area of the space. Gye et al.^13^ proposed a QRA (Quantitative Risk Analysis) method for quantitative risk assessment of high-pressure hydrogen refueling stations in densely populated and highly congested urban areas.

The finite element method is also regarded as the common method for hydrogen leakage problems^14–16^. Numerous experiments and simulations conducted by scholars, focusing on hydrogen accident simulation through computational fluid dynamics software, have provided both valid data and a theoretical foundation for comprehending the mechanism of hydrogen leakage^17–19^. Kuroki et al.^20^ carried out a fire thermal radiation risk assessment study on hybrid hydrogen station. Qian et al.^21^ compared the unintended hydrogen release in both momentum-buoyancy dominated regime and momentum dominated regime. The results show that the profile of the flammable gas cloud goes upward in the momentum-buoyancy dominated regime. The flammable gas cloud is more likely to accumulate towards the ground in a momentum-dominated environment. Li et al.^22^ have conducted a comprehensive analysis of the leakage and diffusion distribution of natural gas and hydrogen mixtures within a closed container through numerical simulations. While their study provides valuable insights into confined environments, it is imperative to extend the investigation to the leakage process in open space. Exploring the dynamics of gas mixture dispersion in unconfined settings will contribute significantly to a more thorough understanding of the overall behavior and potential risks associated with these substances. Liang et al.^23^ employed FLACS software and a computational fluid dynamics approach to simulate hydrogen storage system leakage and explosions in a renewable energy hydrogen production station. The consequences of accidents were analyzed in their study, including the harmful area, lethal area, farthest harmful distance, and longest lethal distance, in relation to variables such as wind velocity, leakage direction, and wind direction. However, there is a lack of necessary suggestions for the structural renovation and optimization of hydrogen station.

The innovation of this paper lies in extending above researches to the leakage process in open spaces. Moreover, a set of optimization suggestions for obstacle and ceiling in the hydrogen stations has been proposed. In this work, the leakage of Hydrogen station in an open space was simulated by finite element method. The distribution of the most unfavorable combustible gas cloud was calculated under no-wind conditions through the leakage of various equipment in the hydrogen station. The effects of different external wind speeds, leakage directions, obstacle structures, and ceiling structures were compared, and the mechanism of leakage and diffusion was analyzed. Finally, preventive measures for hydrogen leakage and explosion accidents in the hydrogen station were proposed. Necessary suggestion has been drawn in the conclusion.

## Method and models

### Governing equation

The FLACS (flame acceleration simulation)^24,25^ is a computational fluid dynamics software adopting the finite volume method. The temperature, fuel concentration, combustion products, overpressure, and other variables in the computational domain can be determined by the semi-implicit method of pressure coupled equations combined with boundary conditions. The governing equations was established based on energy conservation, momentum conservation, mass conservation, and component conservation. The interaction between the flame and overpressure generated by explosion and surrounding environment was considered as following equation:

1 $$\frac{\partial }{\partial t}\left( {\rho \varphi } \right) + \frac{\partial }{{\partial x_{j} }}\left( {u_{i} \rho \varphi } \right) - \frac{\partial }{{\partial x_{j} }}\left[ {\rho \Gamma_{\varphi } \frac{\partial }{{\partial x_{j} }}\left( \varphi \right)} \right] = S_{\varphi } ,$$

where, *φ* is the variable of the conservation equations of mass; momentum, and energy; *ρ* is the density of air, kg/m^3^; ∂*x*_*j*_ represent differential along the *j* direction, m; *u*_*i*_ is the velocity along the *i* direction, m/s; *Γ*_*φ*_ is the diffusion coefficient; *S*_*φ*_ is the source term.

The concentration of gas mixture is determined by the global equivalence ratio *θ*, where the *θ*_act_ represents the actual mass ratio of fuel to oxygen, kg/kg; the *θ*_sto_ represents the stoichiometric mass ratio of fuel to oxygen, kg/kg; *m*_oxygen_ is the mass of oxygen in the gas diffusion zone, kg. The detailed calculation is shown in Eq. (2).

2 $$\theta = \frac{{\theta_{{{\text{act}}}} }}{{\theta_{{{\text{sto}}}} }} = \frac{{\left( {m_{{{\text{fuel}}}} /m_{{{\text{oxygen}}}} } \right)_{actual} }}{{\left( {m_{{{\text{fuel}}}} /m_{{{\text{oxygen}}}} } \right)_{{{\text{stoichiometric}}}} }}.$$

### Virtual nozzle model

**Figure 1:**
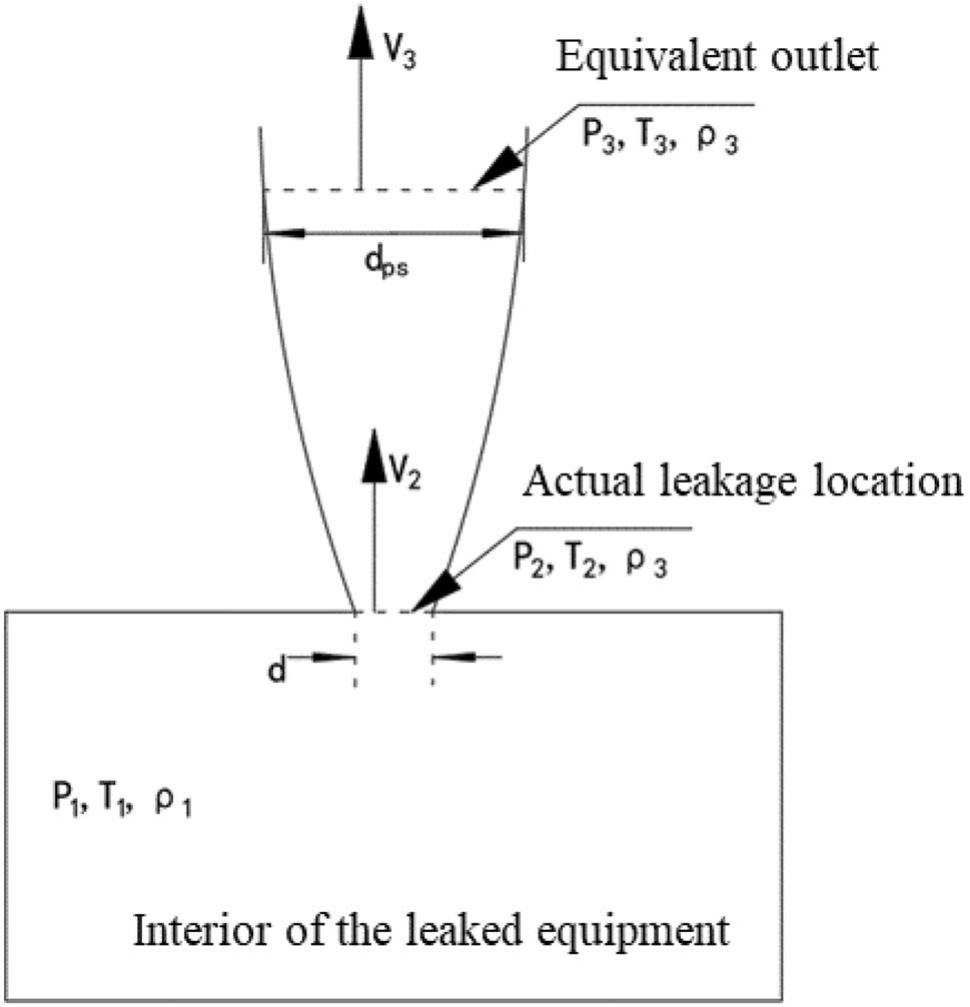
Virtual nozzle model.

A series of excitation force induced by fluid fluctuations at leakage location of high-pressure hydrogen storage tanks, which make the fluid parameters for numerical calculations difficult to converge. Therefore, the virtual nozzle model^26,27^ was used to simplify the study of high-pressure hydrogen storage vessel leakage. The equivalent outlet was substituted for leakage outlet. It should be noted that there is not such a location in the actual leakage process, which is only an assumption made to meet the needs of the model. The calculation principle is shown in Fig. 1, where *P*_1_,* P*_2_,* P*_3_ represent the pressure of interior medium in the hydrogen storage container, leakage location, and the equivalent outlet, Pa;* T*_1_,* T*_2_,* T*_3_ represent the temperature of medium, K; *ρ*_1_,* ρ*_2_*, ρ*_3_ represent the density of medium, kg/m^3^. The pressure of the jet-flow at the equivalent outlet is the environmental pressure. According to the conservation of mass, the flow rate at the equivalent outlet is equal to the flow rate at the actual leakage location. Other flow parameters can be calculated based on the virtual nozzle model.

As depicted in Fig. 1, the mass flow rate at the leakage location *Q*_2_ can be expressed as Eq. (3).

3 $$Q_{2} = \frac{1}{4}\pi d^{2} \rho_{2} V_{2} ,$$

where *V*_2_ local acoustic velocity of hydrogen gas at the actual leakage location, m/s. The mass flow rate at the equivalent outlet *Q*_3_ can be expressed as Eq. (4).

4 $$Q_{2} = \frac{1}{4}\pi d_{ps}^{2} \rho_{3} V_{3} ,$$

where *V*_3_ surrounding environmental local acoustic velocity of hydrogen gas, m/s. According to the continuity equation, the relationship between the parameters at two location is as follows.

5 $$\left( {\frac{{d_{ps} }}{d}} \right)^{2} = \frac{{V_{2} }}{{V_{3} }}\frac{{\rho_{2} }}{{\rho {}_{3}}}.$$

Due to the instantaneous completion of the flow process from hydrogen storage container to actual leakage location, the flow can be regarded as adiabatic flow. Therefore, based on the isentropic relationship equation, the fluid parameters at actual leakage location can be obtained.

6 $$P_{2} = P_{1} \left( {\frac{2}{\gamma + 1}} \right)^{{\gamma /\left( {\gamma - 1} \right)}} ,$$

7 $$T_{2} = T_{1} \left( {\frac{2}{\gamma + 1}} \right),$$

where *P*_2_ is the pressure of actual leakage location, Pa; *T*_2_ is the thermodynamic temperature of actual leakage location, K; *γ* is the specific heat ratio, taken as 1.40. Other fluid parameters were derived from ideal gas state equation.

8 $$\rho_{2} = P_{1} \left( {\frac{2}{\gamma + 1}} \right)^{{1/\left( {\gamma - 1} \right)}} \frac{M}{{RT_{1} }},$$

where *M* is the molar mass of hydrogen; *R* represents the universal gas constant, taken as 8.314 J·mol^−1^·K^−1^.

According to the proportional relationship between local sound velocity and the square root of thermodynamic temperature, the velocity of leakage location and equivalent outlet can be expressed as follow:

9 $$V_{2} = \sqrt {\left( {\frac{{\gamma RT_{2} }}{M}} \right)} = \sqrt {\left[ {\left( {\frac{2\gamma }{{\gamma + 1}}} \right)\frac{{RT_{1} }}{M}} \right]} ,$$

10 $$V_{3} = \sqrt {\left( {\frac{{\gamma RT_{3} }}{M}} \right)} .$$

The length relationship between the actual leakage diameter and the equivalent outlet diameter can be obtained by substituting Eqs. (9) and (10) into Eq. (5).

11 $$\frac{{d_{ps} }}{d} = \sqrt {\left( {\frac{{P_{1} }}{{P_{3} }}} \right)\left( {\frac{{T_{3} }}{{T_{1} }}} \right)\left( {\frac{2}{\gamma + 1}} \right)^{{{{\left( {\gamma + 1} \right)} \mathord{\left/ {\vphantom {{\left( {\gamma + 1} \right)} {2\left( {\gamma - 1} \right)}}} \right. \kern-0pt} {2\left( {\gamma - 1} \right)}}}} } .$$

## Physical model

**Figure 2:**
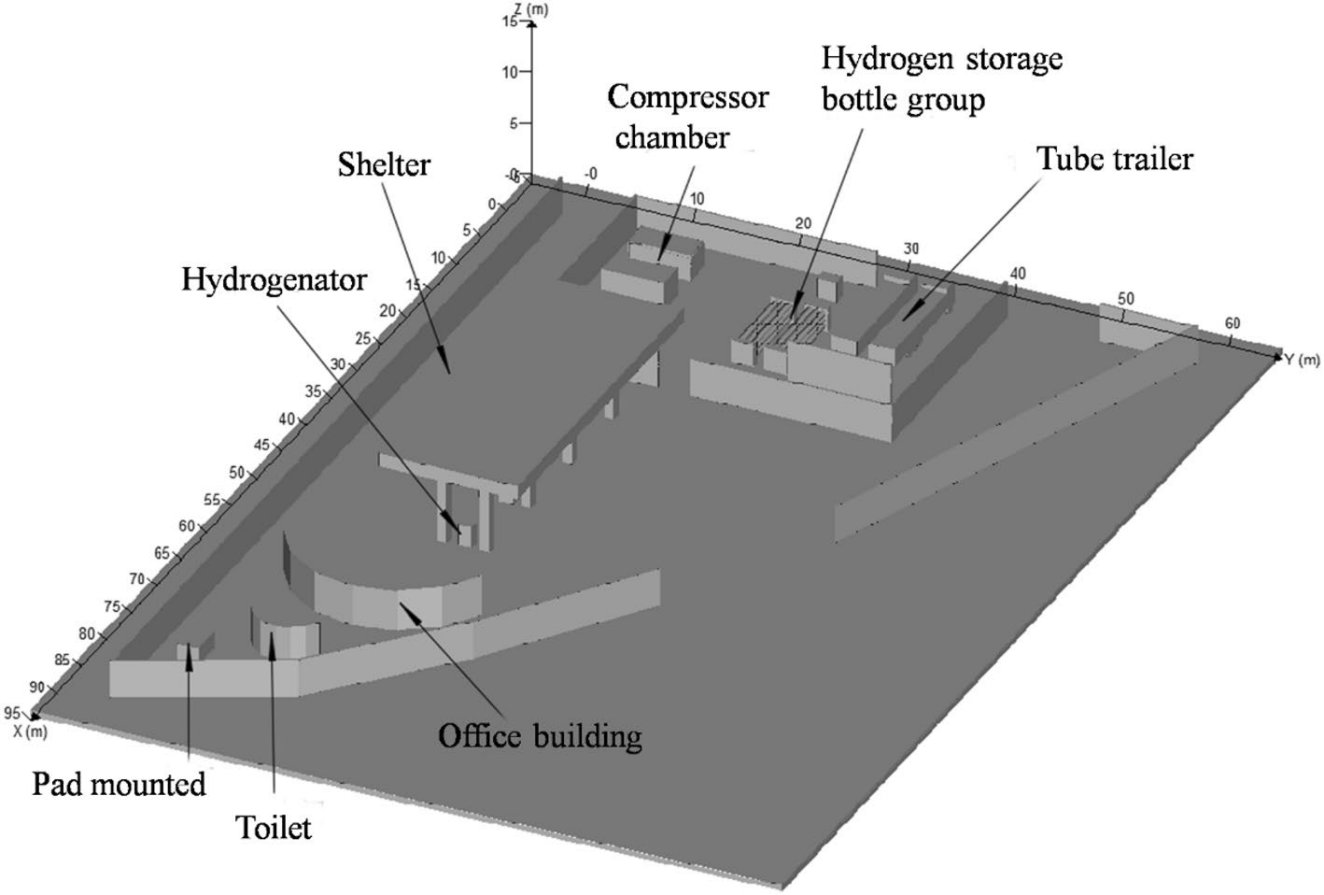
Plane layout of hydrogenation station created by FLACS [version: 9.0; URL: https://www.gexcon.com/support/flacs-cfd/downloads/].

To investigate the impact of different leak locations, leak directions, leakage pressure, and external wind speed on the consequences of accidents, high-pressure hydrogen leakage simulation was carried out. A 1:1 physical model of a hydrogen station was simplified and established in pre-processing module CASD, as shown in Fig. 2. The hydrogen station is generally in the form of a right triangle. The three-dimensional coordinate system of hydrogen station was established centered on the southwest corner. The east direction is the positive direction of the X axis, the north direction is the positive direction of the Y axis, and the vertical direction is the positive direction of the Z axis.

Approximately 90 m from east to west and 60 m from south to north, the west and south sides are occupied by certain public buildings, and the north and south sides by a highway. The station equipment mainly includes 2 hydrogen tube trailers, 2 unloading columns, 2 compressors, 2 fixed gas storage cylinder sets, 4 hydrogenation machines, station buildings, shelters etc.

The pressure of seamless steel gas cylinder during transportation in tube trailer is 20 MPa, while the exhaust pressure is 5 MPa. The volume of gas cylinder is 26 m^3^. The type of hydrogen refueling machine is dual gun, dual metering, and three-line, with a filling pressure of 35 MPa. The compressor model is PDC-13-7500 (100). The specific parameters are shown in Table 1.

**Table 1:** Parameters of compressor PDC-13-7500 (100).

Project	Parameter	Project	Parameter
Gas type	Hydrogen	Number of compressor stage	one stage
Intake pressure	5 MPa-20 MPa	Cooling method	water-cooling
Exhaust pressure	45 MPa	Machine configuration	reciprocating diaphragm
Outlet temperature	≤ 40 °C	Motor power	55 kW
Flow rate	500 kg(12 h) @12.5 MPa	Power supply	AC(380 V), 50 Hz

The parameters of the hydrogen storage bottle group are listed in Table 2.

**Table 2:** Parameters of the hydrogen storage bottle group.

Project	Parameter
Nominal operating pressure	45 MPa
Ambient temperature	− 40 °C–60 °C
Cylinder material	4130X
Hydrogen storage quality	466.42 kg
Number of steel cylinders	9 × 2 groups
Nominal volume of bottle group	0.895m^3^ × 9 × 2 = 16.1m^3^
Filling Medium	Hydrogen
Total length	11 m

According to the formulas (2), (3), (4), (5), (6), (7), (8), (9), (10) and (11), the boundary conditions at the leakage location of the equipment in the hydrogen station can be found in Table 3. The values of temperature were all set to 15 °C, which is determined by ambient temperature.

**Table 3:** Boundary conditions for the leakage location of equipment.

Equipment	Pressure (MPa)	Leakage diameter (mm)	Equivalent diameter (mm)	Mass flow rate (kg/s)	Temperature (°C)
Tube trailer	20	10	107	0.983	15
Compressor	45	10	160	2.211	15
Hydrogen storage bottle group	45	10	160	2.211	15
Hydrogen dispenser	35	10	141	1.719	15

## Results and discussions

### Leakage of hydrogen storage tank group

To simulate the diffusion of hydrogen leakage under the most unfavorable conditions, the pressure at which hydrogen leakage occurred in the hydrogen storage cylinder group was taken as 45 MPa. FLACS v9.0 can only simulate leakage diffusion at a constant leakage rate during simulation, and cannot fully simulate the leakage process of pressure vessels. Therefore, during the simulation setup process, it is assumed that hydrogen gas leaks at a constant velocity calculated at the highest pressure.

**Figure 3:**
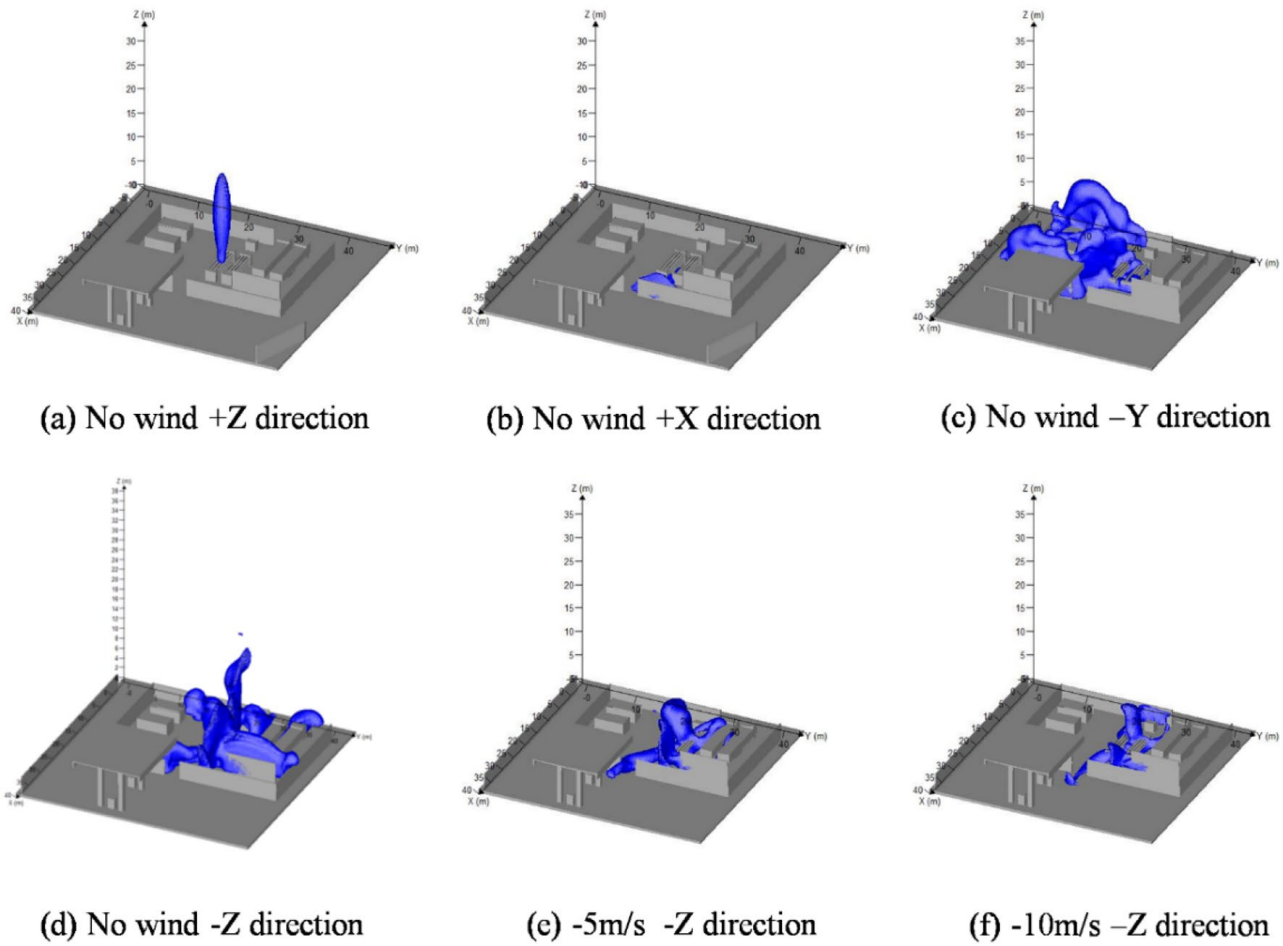
Leakage of hydrogen at the hydrogen storage cylinder group (t = 5 s) created by FLACS [version: 9.0; URL: https://www.gexcon.com/support/flacs-cfd/downloads/].

The leakages of combustible gas under different environmental wind speeds and leakage locations for 5 s were shown in Fig. 3. Distribution of combustible gas leaked from hydrogen storage cylinder group in + Z, + X, -Y, and -Z direction under no wind conditions were depicted as Fig. 3a–d respectively. Under external wind conditions of 5 m/s, Fig. 3e depicted the distribution of combustible gas l that escaped from the hydrogen storage cylinder group in the -Z direction. Under external wind conditions of 10 m/s, distribution of combustible gas was shown as Fig. 3f.

When the leakage was along the + Z direction, the leakage of hydrogen was perpendicular to the ground and upward, with no obstacles or external wind disturbance. It can be found that the combustible hydrogen cloud was in the shape of a vertical and elongated "rugby", as depicted in Fig. 3a. The possibility of an explosion occurring when encountering an ignition source was close to zero since the lack of contact between the leaked gas and any facilities in the station.

When the leakage was along the + X direction, the leakage of hydrogen was squeezed towards the partition wall by high-pressure, as shown in Fig. 3b. The building in the direction of leakage was a partition wall with a simple structure. The hydrogen diffused to all sides as the leaking gas hit the partition wall, accompanied by a loss of kinetic energy during the process. Due to the low density and rapid diffusion of hydrogen, it rapidly diluted upwards, resulting in a smaller volume of combustible gas clouds.

When the leakage was along the -Y direction, the hydrogen leaked from the hydrogen storage bottle group was blocked by another hydrogen storage bottle group, as illustrated in Fig. 3c. After reaching another hydrogen storage bottle group, the high-pressure jet was diffused between the gaps of the hydrogen storage bottles, ultimately forming a large area of irregular combustible hydrogen cloud. However, due to the obstruction of the partition wall, the combustible hydrogen cloud was restricted within the process area.

When the leakage was along the -Z direction, the leakage of hydrogen was perpendicular to the ground and downward, as indicated in Fig. 3d. Under the influence of air buoyancy, hydrogen dispersed across the ground and to nearby facilities or buildings, generating irregular combustible hydrogen cloud. Due to the obstruction of the partition wall, combustible hydrogen clouds were similarly confined within the process area.

It can be found that the influence of hydrogen leakage direction on the shape and volume of combustible hydrogen clouds was mainly determined by the structure and density of obstacles in the leakage direction. If there were no obstacles in the leakage direction or if the structure of the obstacles was regular and simple, the leaked combustible hydrogen cloud had a regular shape and small volume. The probability of explosion when encountering an ignition source is relatively low. On the contrary, if obstacles were densely congested in the leakage direction, the leaked combustible hydrogen cloud had an irregular shape and larger volume, making it more likely to cause an explosion accident.

As Fig. 3e and findicated, the distribution of combustible hydrogen cloud varied from the influence of different velocity of external wind. As the external wind velocity increased, the volume of combustible gas gradually decreased. It can be concluded that the external wind can accelerate the diffusion and dilution of hydrogen in the air. The higher the wind velocity, the stronger the turbulence effect of the atmosphere. The safety of the hydrogen station was guaranteed due to the smaller volume of leaked combustible gas. Even in the presence of external wind, the combustible hydrogen cloud can still be contained within the process area of the hydrogen station, preventing the leaked hydrogen from spreading to other regions.

### Leakage of tube trailer

To further investigated the diffusion of hydrogen leakage under the most unfavorable conditions, the influence of external wind on the other key facilities was neglected. The velocity of external wind was set to zero.

**Figure 4:**
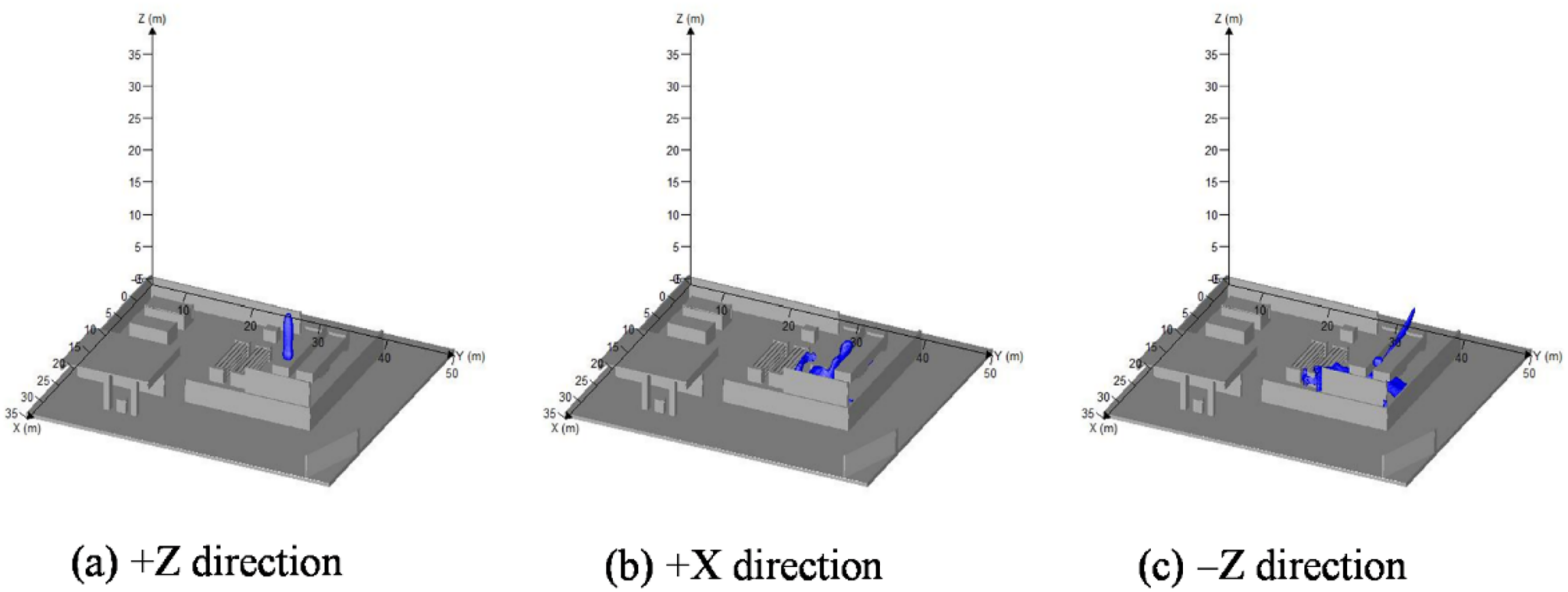
Leakage of hydrogen at tube trailer. (t = 5 s) created by FLACS [version: 9.0; URL: https://www.gexcon.com/support/flacs-cfd/downloads/].

The leakages of tube trailer in different directions for 5 s were indicated in Fig. 4. Distributions of combustible gas leaked from tube trailer in + Z, + X, and − Z direction under no wind conditions were depicted as Fig. 4a–c.

The pressure of hydrogen leakage from the tube trailer was 20 MPa, while from the hydrogen storage tank group is 45 MPa. It can be found that the shapes of combustible hydrogen clouds leaked from tube trailer and hydrogen storage tank group in the same direction were similar. Due to the lower pressure inside the tube trailer, the volume of combustible hydrogen cloud is smaller. Therefore, probability of explosion accidents and damage level caused by explosion accidents were lower.

### Leakage of compressor chamber

**Figure 5:**
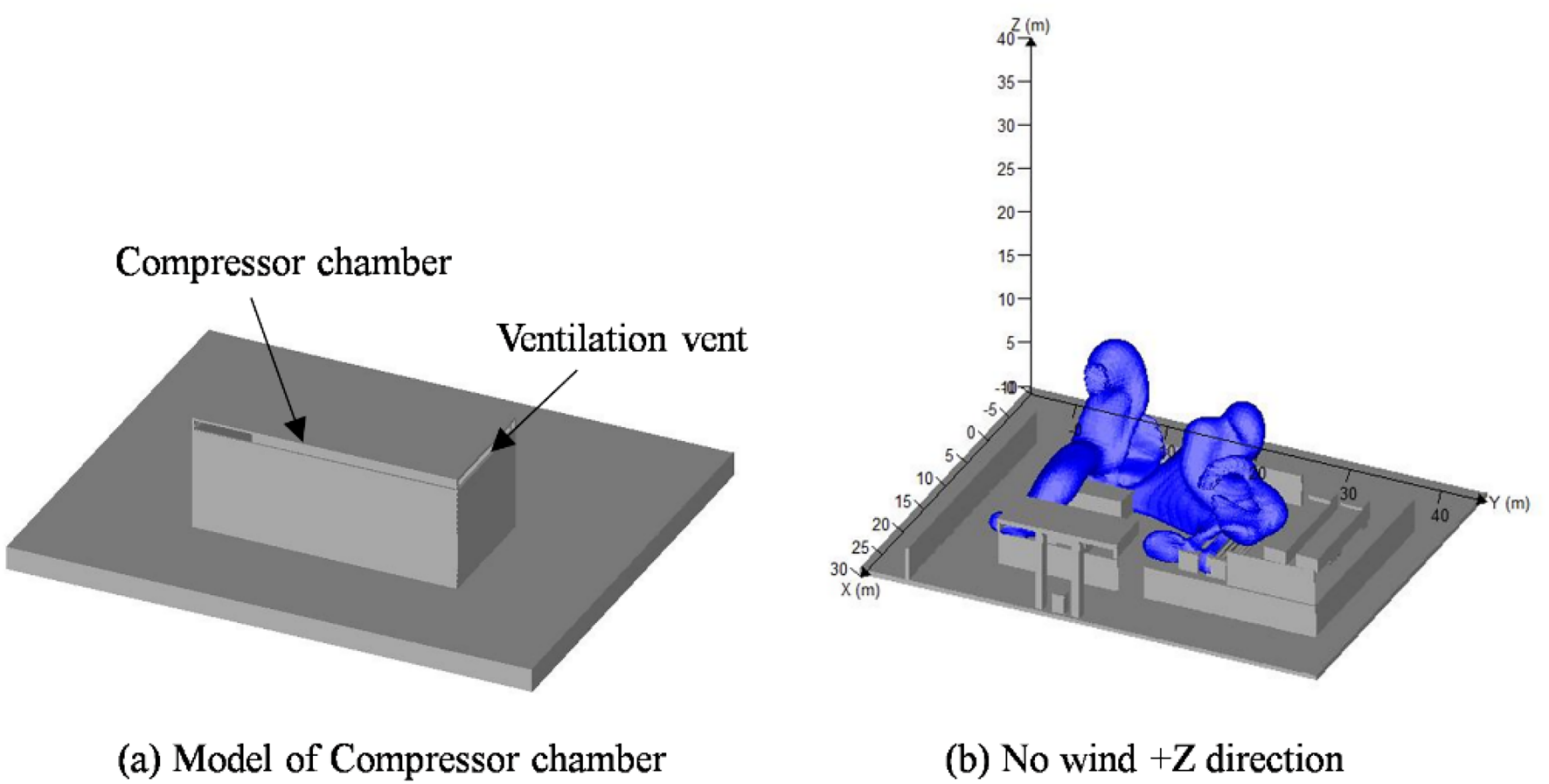
Leakage of hydrogen at compressor chamber. (t = 5 s) created by FLACS [version: 9.0; URL: https://www.gexcon.com/support/flacs-cfd/downloads/].

The physical model of compressor chamber was shown in Fig. 5a. A ventilation vent with a length of 0.3 m was installed above the compressor chamber. In order to simplify the model and computational burden, internal devices such as compressors, pipelines, and valves were ignored. The leakage of compressor chamber in + Z direction for 5 s was indicated in Fig. 5b.

The compressor chamber was a confined space. The leaked hydrogen was initially restricted by the wall before diffusing into the surrounding environment via the vents. As shown in Fig. 5b, the hydrogen escaping from the vent diffused around the compressor chamber. The combustible hydrogen cloud spread to the areas of circulating pumps, tube trailer, and hydrogen storage bottle group. The shape of combustible hydrogen cloud was irregular. Despite its large volume, the combustible hydrogen cloud was still limited by the partition walls in the process area.

### Leakage of hydrogenator

**Figure 6:**
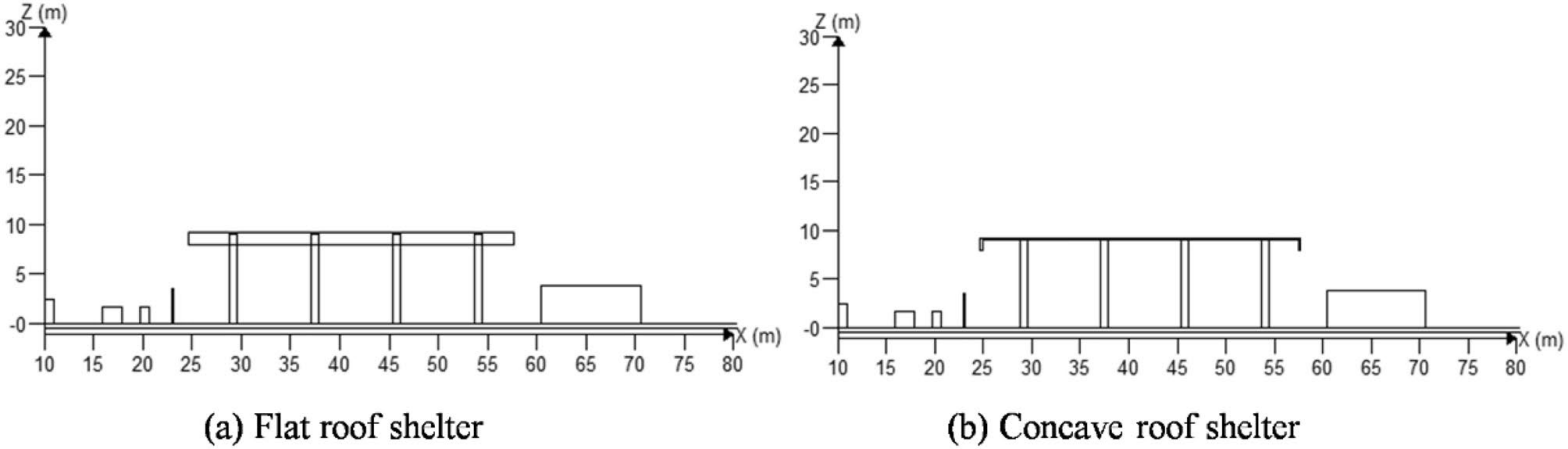
Sectional view of the shelter in the hydrogenation machine area.

Sectional views of hydrogenator with flat roof shelter and concave roof shelter were depicted in Fig. 6a and b respectively. The leakage direction was + Z, the leakage pressure was 35 MPa, and the leakage time was 5 s.

**Figure 7:**
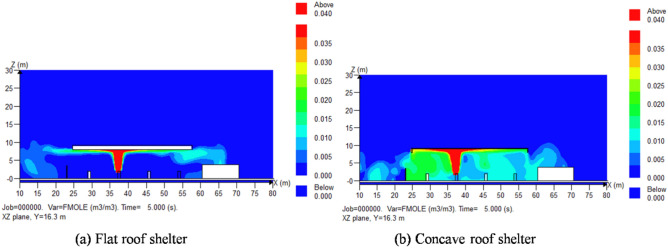
The distributions of hydrogen concentration under different roof shelter at Y = 16.3 m plane.

The distributions of hydrogen concentration under different roof shelter at Y = 16.3 m plane were shown in Fig. 7. Areas with hydrogen concentrations over the lower explosive limit (4%) were marked in red. The shapes of red area in the two types of shelter were similar, which both were beneath the shelter and above the hydrogenator. Therefore, the probability of explosion occurring when encountering an ignition source is relatively low.

The high-pressure hydrogen jet leaking in the + Z direction diffused along the bottom of the hydrogenator shelter. Along the flat roof shelter, hydrogen diffused into the atmosphere via bottom surface of shelter and gradually diluted upwards. Along the concave roof shelter, hydrogen was obstructed by the vertical planes around the shelter, causing it to diffuse to the area between the ground and the shelter. The hydrogen concentration inside the area was around 2%. The concentration was below the lower explosion limit. However, if there was a problem of filling vehicles or sundries storage in the hydrogenator, the hydrogen concentration in the area will be increased by the poor ventilation. In addition, long-term leakage may also cause the local concentration of hydrogen to reach the lower explosion limit. As a result, using a flat roof shelter in a hydrogenator is safer than using a concave roof shelter.

## Conclusions

In this paper, the diffusion of hydrogen leakage under the most unfavorable conditions was simulated by finite element analysis method. The main conclusions are drawn as follows:

1. The distribution shape of leaked hydrogen was mainly determined by the structure and density of obstacles in the leakage direction. The dense blockage of obstacles in the direction of leakage can cause the diffusion of combustible hydrogen clouds to be irregular in shape, larger in volume, and more likely to cause explosion accidents. Optimizing equipment layout, minimizing obstacles in the direction of leakage should be considered to ensure smooth ventilation systems
2. The device with higher hydrogen storage pressure produces a larger volume of combustible hydrogen gas cloud in the event of a leakage. Safety measures should be categorized based on the hydrogen storage pressure of different devices.
3. The turbulence effect caused by external wind has a positive effect on the dilution of leaked gas. As the external wind velocity increased, the volume of combustible gas gradually decreased.
4. Prioritize choosing a flat roof shelter over a concave roof shelter. The combustible gas diffusion performance of flat roof shelter is better than that of concave roof shelter. A large amount of combustible gas will accumulate inside the concave roof shelter, which will cause potential safety hazards to the hydrogenator.

## Data Availability

The datasets used and/or analysed during the current study available from the corresponding author on reasonable request.
